# Recurrent prostatic stromal sarcoma (PSS): A case report and literature review

**DOI:** 10.1016/j.eucr.2025.103118

**Published:** 2025-07-04

**Authors:** Mohammad Reza Nowroozi, Mohammad Hamidi Madani, Amirreza Farzin, Saeed Montazeri, Zahra Tahamtani Torkamani, Ehsan Hajiasadi

**Affiliations:** a Uro-Oncology Research Center, Tehran University of Medical Sciences, Tehran, Iran; b Faculty of Medicine, Tehran University of Medical Sciences, Tehran, Iran; c Student Research Committee, Qazvin University of Medical Sciences, Qazvin, Iran

**Keywords:** Prostatic stromal sarcoma, Mesenchymal tumor, Histopathology, Pelvic mass, Magnetic resonance imaging, Hematuria

## Abstract

Prostatic stromal sarcoma (PSS) is an exceptionally rare mesenchymal tumor of the prostate. We present a case of high-grade recurrent PSS in a 49-year-old man, managed with complete surgical excision and successful organ preservation. This report is accompanied by a literature review highlighting diagnostic challenges, histopathological features, and evolving surgical approaches, including robotic-assisted techniques. By comparing our case with previously reported cases, we emphasize the importance of individualized treatment strategies and long-term follow-up in managing this aggressive malignancy.

## 1 Introduction

Malignant stromal tumors, unlike prostate adenocarcinomas, account for only 0.1–0.2 % of all prostate cancers.^1^ Prostatic stromal tumors are subcategorized into prostatic stromal sarcoma (PSS) and stromal tumor of uncertain malignant potential (STUMP).^2^ Although the term STUMP encompasses cases that may potentially be benign, these tumors are considered a neoplastic entity due to their capacity to recur, extensively infiltrate the prostate gland with potential extension to adjacent tissues, and progress to prostatic stromal sarcoma (PSS) with the possibility of distant metastasis.^3^ At first, prostate stromal tumors were classified into 2 types: prostate stromal sarcoma (PSS) and STUMP. Later on, Mokhtari et al. found that the prevalence of STUMP in patients diagnosed with benign prostate hyperplasia was 0.43 %.^4^. Due to its origination from nonepithelial mesenchymal tissues such as smooth muscle, blood vessels and nerves, prostate sarcoma can manifest as different pathological types.^5^ The age of prostatic stromal tumor patients varies from 23 to 81 years, with more occurrence in the ages of 50–70.^6^ Clinical presentation varies and may include lower urinary tract symptoms, elevated prostate-specific antigen (PSA) levels, hematuria, abnormal findings on digital rectal examination, and rectal obstruction.^3^ A definitive diagnosis relies on postoperative histopathological examination. While histopathology primarily determines whether a tumor is benign or malignant, additional techniques such as immunohistochemistry are essential to accurately identify the tumor's origin and to provide a specific diagnosis.^7^ There are fewer than 100 cases reported as PSS, which makes it a rare condition.

## 2 Case presentation

We present the case of a 49-year-old man who initially presented to our hospital in 2022 for further evaluation of chronic pelvic pain and a single episode of gross hematuria. Comprehensive investigations revealed no underlying systemic disease or relevant family history. Digital rectal examination (DRE) identified prostatic adenomatosis of moderate size and adenomatosis without nodule Serum prostate-specific antigen (PSA) was reported at 4.6 ng/mL.

**Fig. 1:**
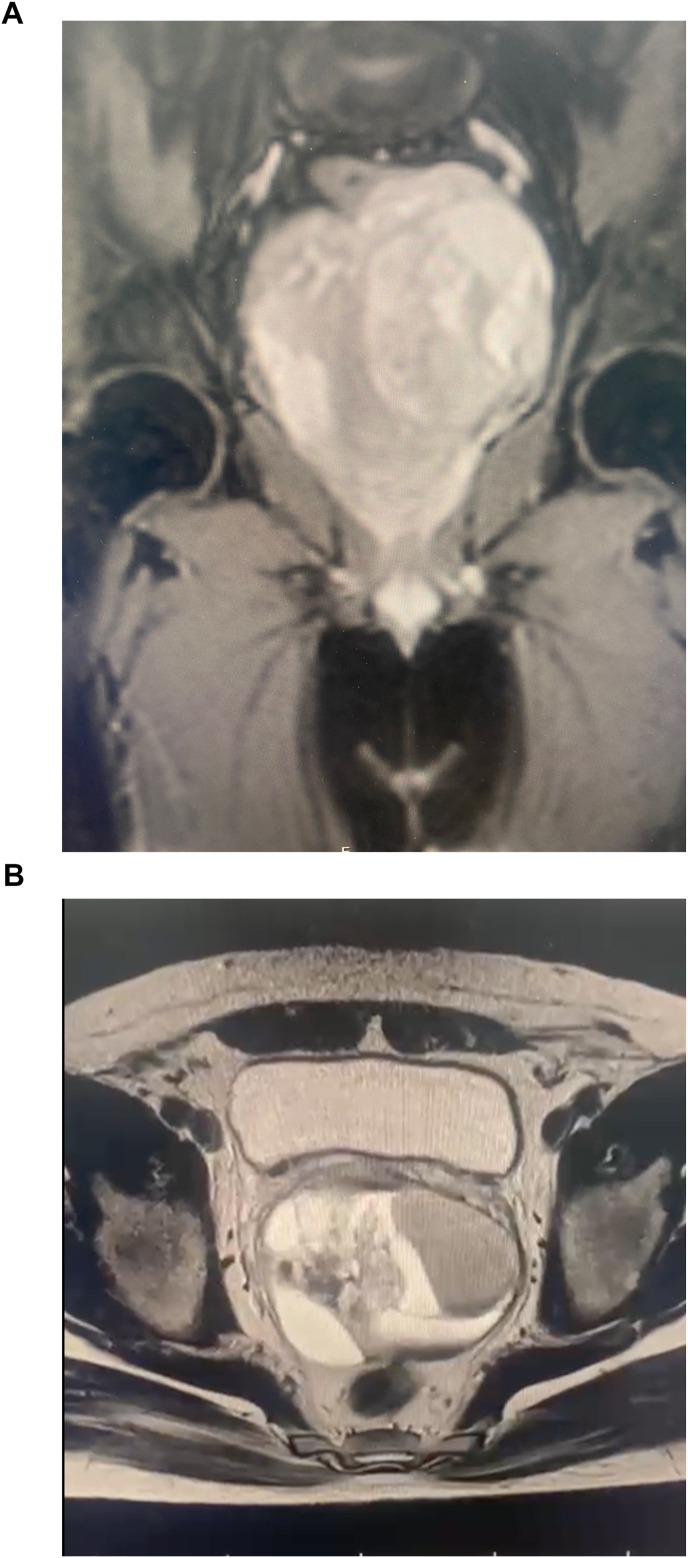
Pelvic MRI of the multilocular cystic mass. **a** Coronal T2-weighted image showing a large multilocular lesion located posterosuperior to the prostate, causing displacement of the rectum and bladder. **b** Axial T2-weighted image demonstrating heterogeneous signal intensity with internal septations and compression of adjacent pelvic organs.

Subsequent sonography and MRI revealed a 107 × 87 mm multiseptated, multilocular cystic mass located posterosuperior to the prostate, exerting significant compression on the rectum and bladder. The mass exhibited multiple enhancing thick septa and areas of hemorrhage. Surgical excision of the cystic lesion was performed. (Fig. 1).

According to paraclinics and physical exam the patient was considered for surgery and the patient was operated on with the preoperative assumption of a hemorrhagic cyst. A lower midline incision was made, and the abdominal cavity was entered via an intraperitoneal approach. Intraoperatively, a mass associated with a hematoma was observed in the space between the bladder and the right pelvic wall. The hematoma was evacuated, and the residual cystic components were resected. Gross pathological examination of the excised specimen, received in formalin, described a pale tan soft tissue fragment measuring 2.2 × 1.3 × 1.5 cm with a smooth internal surface. Histopathological analysis identified a congested cyst wall lacking a true epithelial lining, with focal infiltration by chronic inflammatory cells. The lesion was designated as a pelvic wall cyst.

Since that time until 2024, no further follow-up was conducted. However, in 2024, the patient presented to our hospital once more, with urinary frequency, chronic pelvic pain and one episode of gross hematuria. Sonographic evaluation revealed a round, heterogeneous mass measuring 99 × 95 × 75 mm, with a volume of 297 cc. The lesion contained cystic regions with subtle necrosis and demonstrated no apparent vascularity. A rising PSA trend was observed, reaching 30 ng/mL. DRE identified a palpable lesion.

MRI of the prostate demonstrated a gland volume of 8 cc (dimensions: 40 × 21 × 16 mm). However, a large heterogeneous cystic-solid mass measuring 460 cc was identified arising from the posterior aspect of the peripheral zones at the prostate base. The mass exhibited severe necrosis, areas of diffusion restriction, and heterogeneous enhancement, raising strong suspicion for prostatic carcinoma (PIRADS V). Large T1 hyperintensities suggestive of hemorrhage were noted within the lesion. The mass exerted significant pressure on the rectum with suspicious areas of local invasion and caused anterior displacement of the urinary bladder. No lymphadenopathy was identified.

**Fig. 2:**
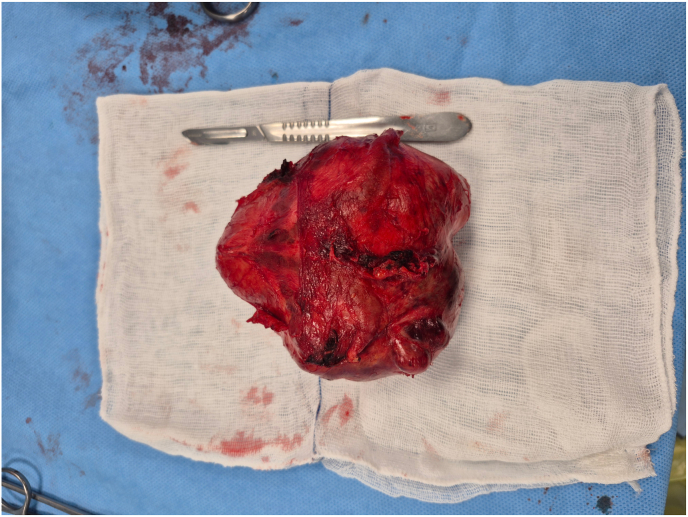
Gross photograph of the resected pelvic mass showing a large, encapsulated, multilobulated lesion with areas of hemorrhage and necrosis.

**Fig. 3:**
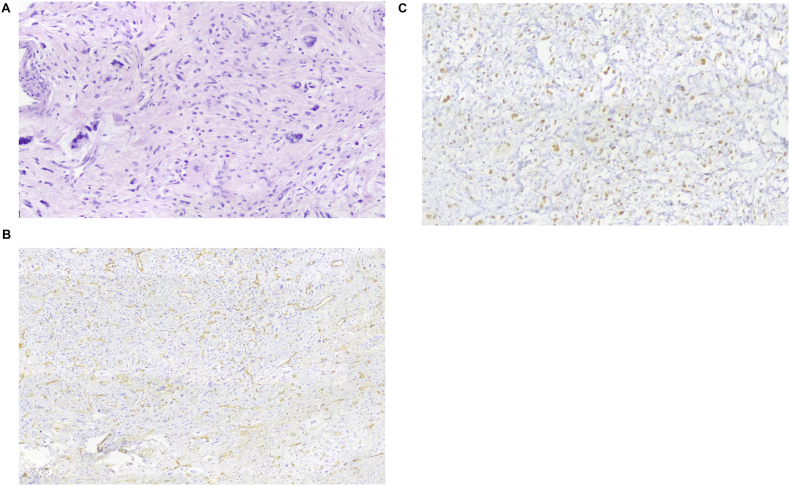
(a) Hematoxylin and eosin (H&E) staining shows spindle cell proliferation with moderate atypia in a myxoid stroma. (b) CD34 immunostaining highlights vascular structures within the tumor. (c) Progesterone receptor (PR) staining shows diffuse nuclear positivity in tumor cells.

A biopsy was performed, initially revealing benign prostatic hyperplasia (BPH). However, targeted biopsy of the lesion confirmed the diagnosis of prostatic stromal sarcoma (PSS). Surgical intervention was planned, and the mass was excised with preservation of the prostate and bladder, given the patient's young age and desire to maintain sexual function. (Fig. 2). Postoperatively, the patient's PSA levels returned to normal, indicating effective local tumor control and supporting the success of the surgical intervention. Surgical pathology reports a large well-confined solid mass of soft tan to gray tissue, measuring 13 × 10 × 4 cm. Microscopic sections show a high-grade stromal tumor, composed of isolated tumor cells having large atypical bizarre nuclei, situated among prostatic glandular structures. (Fig. 3).

## 3 Discussion

Our case represents a rare instance of prostatic stromal sarcoma (PSS), a prostate malignancy that constitutes less than 0.1 % of all prostate malignancies.^1^ PSS is an aggressive and rare form of cancer that typically arises from mesodermal tissue in the reproductive tract with no specific risk factor.^8^. The rarity of PSS significantly hampers the ability to conduct large scale clinical studies, resulting in a paucity of comprehensive data to guide evidence-based treatment strategies and leads to diagnostic delays as the clinical presentation closely mirrors more common urological disorders, such as benign prostatic hyperplasia (BPH).^9^. This case highlights a unique instance of PSS in a 49-year-old man who initially presented with chronic pelvic pain and single episode gross hematuria. Extensive investigation failed to identify any underlying systemic conditions or significant history contributing to the patient's presentation. His serum prostate-specific antigen (PSA) levels, although mildly elevated, did not raise immediate suspicion for malignancy at his first encounter. This case underscores the importance of considering rare entities like PSS in differential diagnoses, especially in patients presenting with common urinary tract symptoms such as hematuria.

Prostate sarcomas encompass various histological subtypes, including leiomyosarcoma, rhabdomyosarcoma, malignant fibrous histiocytoma, and unclassified sarcomas.^8^. Rhabdomyosarcoma is the most common soft-tissue sarcoma in pediatric population, whereas leiomyosarcoma is more frequently diagnosed in adults and represents approximately 30 % of all prostate sarcoma.^10^. Evidence from a published study suggests that patients with leiomyosarcoma exhibit poorer survival outcomes compared to those with non-leiomyosarcoma subtypes.^11^. Unclassified sarcomas can be further subclassified into primary stromal sarcomas and stromal tumors of uncertain malignant potential, based on the degree of mesenchymal overgrowth, the extent of local invasion, and the level of mitotic activity.^12^. To date, a case of primary prostatic stromal sarcoma occurring concurrently with prostate adenocarcinoma has been reported.^13^. Histological examination is crucial for distinguishing between benign and malignant mesenchymal tumors. Malignancy is typically indicated by sever nuclear atypia, a high mitotic rate, atypical mitoses, tumor necrosis, lymphovascular invasion, and local organ invasion. However, histological patterns and cytological morphology may not always provide definitive tumors regarding the tumor's origin. For instance, smooth muscle derive tumors like leiomyosarcoma may be indistinguishable from other spindle cell neoplasms, such as rhabdomyosarcoma, malignant peripheral nerve sheath tumors (MPNST), or vascular tumors like angiosarcoma. Literature suggests that PSSs are consistently Vimentin-positive as in our case, with variable expression of CD34. Additionally immunoreactivity for markers such as SMA, Desmin, Estrogen Receptor (ER), Progesterone Receptor (PR), and in some cases epithelial markers like Pan-cytokeratin (PanCk), has been reported, also these findings remain rare.^14^. Based on targeted biopsy findings, the immunohistochemical profile and histopathological features support the diagnosis of high-grade PSS in patient. The tumor exhibits positivity for Vimentin, PR, and SMA, with negativity for PanCk, S100, Desmin, PSA, CK5/6, BCL2, MDM2, and Melan A. Additionally, the Ki-67 proliferation index is approximately 25 %, and CD34 highlights only the vascular walls without tumor cell positivity. These findings are characteristic of high-grade PSS. The Vimentin and PR positivity align with the mesenchymal origin and hormonal responsiveness of PSS. While CD34 positivity in tumor cells is commonly reported in PSS, its absence does not exclude the diagnosis, as CD34 expression on tumor cells can be variable.^14^ The absence of Desmin and S100 helps exclude leiomyosarcoma and nerve sheath tumors, respectively.^15^. PanCk and PSA negativity rule out prostatic adenocarcinoma, and Melan A negativity exclude out melanocytic tumors.^14^.

Generally, PSS has a similar morphology to STUMP. According to Pan et al.^16^ distinct genomic differences exist between PSS and STUMP. While both tumor types exhibit chromosomal losses on chromosomes 13 and 14, high grade PSS demonstrates a markedly more disordered genomic landscape. Specifically, PSS harbors a significantly higher tumoral mutational burden. Furthermore, high grade PSS is characterized by numerous structural rearrangements and chromosomal copy number alterations, correlating with its clinically aggressive and anaplastic behavior. Differential diagnosis of STUMP and PSS is mainly dependent on histopathological manifestations and IHC markers.^17^

There is no standardized guideline or definition for MRI imaging specific to prostate sarcoma, and the presence of a tumoral lesion is often inferred based on abnormal signal characteristics.^18^. However Imaging modalities particularly MRI (mpMRI) played a pivotal role in delineating the mass. While initial sonography and MRI suggested a large, multilocular mass with internal hemorrhage, which mimicking a benign pelvic cyst, continued symptomatology and mass progression warranted targeted biopsy. MRI revealed a distinct 460 cc heterogenous cystic solid mass with necrosis, hemorrhage (T1 hyperintensities), and PIRADS V features, displacing adjacent structures such as the rectum and bladder. Importantly features such as T2 hypointense pseudo-capsule, central necrosis, and fibrotic perilesional ring favored a diagnosis of sarcoma over adenocarcinoma. Additionally, MR spectroscopic imaging can provide valuable biochemical insights by showing a markedly elevated choline-to-citrate ratio, distinguishing malignant stromal tumors from benign processes like BPH. The targeted prostate biopsy, ultimately confirmed the diagnosis, reinforcing the essential role of histopathologic assessment when imaging is equivocal.^18^ This case underscores the importance of a high index of suspicion and a multimodal diagnostic approach, particularly when clinical features deviate from classic urological pathologies.

Complete surgical excision remains the cornerstone of treatment for prostatic stromal sarcoma, particularly in cases without metastatic spread. Due to its rarity, optimal management strategies are not standardized, but current literature supports radical prostatectomy or cystoprostatectomy as the most effective curative approach for localized disease.^19^ Robot-assisted laparoscopic radical prostatectomy (RLRP) has emerged as a feasible option in select patients, especially when bladder invasion is absent and organ preservation is prioritized. The advantages of RLRP include enhanced visualization, improved precision in dissection, and reduced risk of complications in the confined pelvic space. However, regardless of the surgical approach, achieving negative margins is crucial, as positive margins are associated with a higher risk of local recurrence and poor outcomes.^19^ In our case, the mass was successfully excised, and the prostate and bladder were preserved. (The decision to preserve these structures is due to well-confined nature of the tumor, as seen on imaging and intraoperative findings.) However, despite to successful surgery, PSS has a potential for local recurrence. The patient's rising PSA levels at the time of recurrence raised concern for future malignant progression. This underscores the need for long-time follow up and monitoring, may facilitate timely intervention.

## 4 Conclusion

Prostatic stromal sarcoma is a rare and aggressive malignancy with nonspecific clinical and radiological features that often mimic benign conditions. Accurate diagnosis requires a combination of advanced imaging, histopathology, and immunohistochemistry. Surgical excision remains the cornerstone of treatment, with organ-preserving approaches feasible in select cases. Through this case and accompanying literature review, we highlight the importance of individualized surgical planning and long-term surveillance, as well as the growing role of minimally invasive techniques such as robotic-assisted surgery in optimizing oncologic and functional outcomes.

The reporting of this study conforms to the CARE guidelines.^20^

## CRediT authorship contribution statement

**Mohammad Reza Nowroozi:** Writing – review & editing, Writing – original draft, Visualization, Validation, Supervision, Software, Resources, Project administration, Methodology, Investigation, Funding acquisition, Formal analysis, Data curation, Conceptualization. **Mohammad Hamidi Madani:** Writing – review & editing, Writing – original draft, Visualization, Validation, Supervision, Software, Resources, Project administration, Methodology, Investigation, Funding acquisition, Formal analysis, Data curation, Conceptualization. **Amirreza Farzin:** Writing – review & editing, Writing – original draft, Visualization, Validation, Supervision, Software, Resources, Project administration, Methodology, Investigation, Funding acquisition, Formal analysis, Data curation, Conceptualization. **Saeed Montazeri:** Writing – review & editing, Writing – original draft, Visualization, Validation, Supervision, Software, Resources, Project administration, Methodology, Investigation, Funding acquisition, Formal analysis, Data curation, Conceptualization. **Zahra Tahamtani Torkamani:** Writing – review & editing, Writing – original draft, Visualization, Validation, Supervision, Software, Resources, Project administration, Methodology, Investigation, Funding acquisition, Formal analysis, Data curation, Conceptualization. **Ehsan Hajiasadi:** Writing – review & editing, Writing – original draft, Visualization, Validation, Supervision, Software, Resources, Project administration, Methodology, Investigation, Funding acquisition, Formal analysis, Data curation, Conceptualization.

## Informed consent

Written informed consent was obtained from the patient for publication of this case report and any accompanying images.

## Ethics approval

Not applicable.

## Data availability statement

No datasets were generated or analyzed during the current study.

## Declaration of generative artificial intelligence (AI) and AI-assisted technologies in the writing process

During the preparation of this work, the authors utilized AI **(**Grammarly.com) to refine grammar points and language style in writing. Subsequently, the authors thoroughly reviewed and edited the content as necessary, assuming full responsibility for the publication's content.

## Funding

No external funding was received for this study.

## Conflict of interest

The authors declare no conflicts of interest.
